# Antibodies as Diagnostic Targets and as Reagents for Diagnostics

**DOI:** 10.3390/antib9020015

**Published:** 2020-05-18

**Authors:** Nicole H. Trier, Gunnar Houen

**Affiliations:** Department of Neurology, Rigshospitalet Glostrup, Valdemar Hansens vej 13, 2600 Glostrup, Denmark; nicole.hartwig.trier@regionh.dk

Antibodies (Abs) were discovered around the turn of the 19th century and characterized in the following decades as an essential part of the human adaptive immune system [1,2]. Abs are produced in response to infections with pathogenic organisms and therefore have diagnostic potential. The levels of specific Abs reflect the burden of infection and the type of Abs produced may reflect the duration of infection and the site of infection, since Abs undergo class switching and affinity maturation in the course of infections [1,2]. Initially IgM is produced, but switching to IgG, IgA or IgE occurs during infections, depending on the type of pathogen and the site of infection.

The use of Abs as targets of diagnostics can roughly be divided in five areas:

## 1. Infections

The specificity, type and concentration of Abs have diagnostic value. The specificity giving information on the type of pathogen, the type and concentration giving information on the state of infection (IgM as an indication of early infection, IgG and IgA as indications of prolonged or chronic infections (IgA further indicating mucosal/epithelial infection)) [2].

## 2. Autoimmune Diseases

The specificity, type and concentration of auto-Abs have diagnostic value. The specificity and the type giving information on the tissue/organ involved (IgA indicating mucosal/epithelial affection, IgM being relevant in a few conditions), the concentration giving some information on the degree of affection [3].

## 3. Allergies

The specificity and the concentration of IgE have diagnostic value [4].

## 4. Immune Deficiencies

The type and concentration of Abs missing or being decreased have diagnostic value (genetic or acquired inabilities to produce certain types of Abs cause immune deficiencies, characterized by the type of Ab deficiency and being associated with characteristic types of infections) [2,5].

## 5. Cancer

The occurrence of excessive amounts of certain (monoclonal) Abs or fragments thereof (M components) in certain hematological malignancies and premalignancies have diagnostic value [6].

The five areas show some degree of overlap as immune deficiencies are associated not only with infections but also with some autoimmune diseases and as allergies may have relations with infections [7,8,9].

Characteristic for areas 1–3 is that Abs are the targets of diagnostics and that antigens are used to measure the Abs. In area 4, it is the absence of certain Abs that has diagnostic value. In area 5, it is the excessive production of Abs or fragments thereof that is used diagnostically. Furthermore, in rare cases, which actually belong to area 2, characteristic (paraneoplastic) auto-Abs have immense diagnostic value for some cancers [10].

Due to the very high specificity of Abs and the development of highly effective methods for Ab production and characterization, they are also extensively used as reagents for the measurement of different targets (analytes).

The use of Abs as reagents for detection and quantification of analytes can be divided according to the type of Ab, the assay type, and the analytes measured:

A. The type of Ab can be “polyclonal” (monospecific/polyspecific serum or purified immunoglobulin preparation), monoclonal, a peptide Ab or a recombinant Ab. Moreover, Ab fragments can be used for various assays [11,12,13,14,15].
B. The assay type can be a solution assay (e.g., nephelometry/turbidometry), an electrophoretic assay (e.g., a mobility shift assay or various types of immune-electrophoresis) or solid phase assays (“direct” assays (Ab capture), capture assays (antigen capture), “sandwich” assays, inhibition assays, etc.). Some assay types can be performed as “label-free” (e.g., based on fluorescence-energy transfer, surface plasmon resonance, acoustic), but most assays rely on various detection methods, which can be enzymatic (enzyme-linked immune-sorbent assays (ELISAs)), fluorescence, chemiluminescence, and others. In addition, various combinations of assays are frequently used such as immuno-precipitation, immuno-blotting, line/array-blotting, lateral flow assays, and others. The use of Abs for immunocytochemistry/histochemistry is particularly valuable both for diagnostics and for research [3,6,11,12,13,14].
C. The analytes measured can be virtually any molecule/antigen to which an antibody can be made (hormones, growth factors, vitamins, drugs, etc.), including various Abs and auto-Abs [3,6,11,12,13,14].

In all the above-mentioned assays, non-specific binding and possible interference from rheumatoid factors must be taken into account and corrected for, an issue which is particularly important in diagnostic uses of Abs, since false positive/negative results may lead to wrong diagnoses and treatments [16,17].

This Special Issue of *Antibodies* gives examples of current “state-of-the-art” uses of Abs as targets of diagnostics (infections and autoimmune diseases) [18,19,20,21,22] and as reagents for measuring analytical targets [23,24].
